# *In vitro* combination therapy using low dose clotrimazole and photodynamic therapy leads to enhanced killing of the dermatophyte *Trichophyton rubrum*

**DOI:** 10.1186/s12866-014-0261-z

**Published:** 2014-10-15

**Authors:** C Oliver Morton, Mousawi Chau, Colin Stack

**Affiliations:** School of Science and Health, University of Western Sydney, Campbelltown Campus, Narellan Road, Campbelltown, NSW 2560 Australia

**Keywords:** Photodynamic therapy, *Trichophyton rubrum*, Antifungal therapy

## Abstract

**Background:**

Superficial infections of the skin and mucous membranes caused by dermatophyte fungi are amongst the most common and challenging infections to treat. Previously we demonstrated the phototoxic effects of photodynamic therapy (PDT) towards *Trichophyton rubrum*, using a green laser to photoactivate Rose Bengal (RB). The aim of this study was to evaluate whether we could; (1) achieve a similar effect using an inexpensive light-emitting diode (LED) to photoactivate RB and (2) to evaluate whether our PDT regime could be combined with standard antifungal drug therapy and increase its effectiveness.

**Methods:**

We designed and built our own inexpensive green (530 nm) LED source and tested its efficacy as part our RB-PDT regime *in vitro* against *T. rubrum*. We also examined the potential benefits of incorporating PDT as part of combination therapy and whether the order in which this was done had an impact. First we subjected spore suspensions to sub-inhibitory concentrations of a number of antifungal agents (CLT, MCZ and TRB) for 72 hours followed by RB-PDT. Secondly we subjected spore suspensions to sub-inhibitory PDT followed by drug treatment and evaluated if there were any changes to the minimum inhibitory concentrations (MICs) of the drugs tested.

**Results:**

The optimal conditions for photoinactivation of *T. rubrum* using RB-PDT alone were 140 μM of RB and 24 J/cm^2^ of LED (equating to a 30-minute exposure). These parameters also caused a 100% reduction in the viability of the pathogenic yeast *Candida albicans* and the model fungus *Saccharomyces cerevisiae*. By combining our RB-PDT regime as an adjunct to antifungal drugs we were able to dramatically reduce the exposure times. Treatment of spore suspensions using a sub-inhibitory dose of clotrimazole (CLT) followed by RB-PDT, this order was critical, significantly reduced the exposure times required to achieve 100% inhibition of *T. rubrum* to 15 minutes as compared to RB-PDT alone.

**Conclusions:**

The combination of antifungal drug and RB-PDT represents an attractive alternative to the current antifungal therapies used to treat superficial fungal diseases. Our approach has the potential to reduce treatment times and drug dosages which can also reduce drug toxicity and improve patient compliance.

**Electronic supplementary material:**

The online version of this article (doi:10.1186/s12866-014-0261-z) contains supplementary material, which is available to authorized users.

## Background

Dermatophytic fungi cause a range of superficial diseases, termed dermatophytoses, which affect keratinised tissues of humans such as the skin, hair and nails [1]. Fungal infections of the skin and nails are easily spread and have a worldwide distribution. However, the incidence and severity tends to be greater in those with compromised immune systems [2]. Individuals such as the young and old or those suffering from underlying conditions such as HIV, *diabetes mellitus* and cancer patients undergoing chemotherapy are at greatest risk [2,3]. Furthermore, fungal infections can be very challenging to treat with high recurrence rates [4].

Treatment of fungal infections involves either the administration of topical or oral antifungal agents (systemic) or a combination of both depending on the area infected (e.g. skin or nails) [5,6]. For treatment of superficial infections, topical medications containing the antifungal agent clotrimazole are the most commonly used [7,8]. While more difficult to treat infections such as those affecting the toenails (termed onychomycosis) are treated using systemic antifungals agents such as terbinafine [9].

Both fungi and their human hosts are eukaryotes which limits the number of drugs that display selective toxicity [10]. Some antifungals with overlapping toxicity must be used at low doses, which causes some fungal infections to require extended treatment times of up to 12 months [11]. Patient non-compliance is a major issue leading to abandonment of treatment, which in turn contributes to high rates of recurrence. Abandonment can also be due to the high costs associated with ongoing treatment [12]. Furthermore, the long-term use of antifungals is contra-indicated in some patients, particularly the elderly, who may experience drug-drug interactions with other medications, and in patients with abnormal liver function [13].

We believe that the ideal antifungal therapy should be fungicidal in its mode action (actually kills the fungus), of short duration, easy to take/deliver, have minimal adverse side-effects, and importantly be affordable. Photodynamic therapy (PDT) offers an attractive alternative to the use of antifungal drugs, negating many of the aforementioned treatment issues. PDT is a non-invasive clinical therapy currently being utilised in the treatment of skin tumours [14]. PDT involves the use of visible light (usually a laser), a non-toxic photosensitiser (PS) or light activated dye and the presence of molecular oxygen [15,16]. Individually, these various components are harmless. However, once activated by the light source the PS becomes energised to an excited state reacting with molecular oxygen to generate reactive oxygen species (ROS). The presence of these toxic ROS leads to the oxidation of biomolecules that ultimately kill target cells [17-19]. We have previously demonstrated the antifungal effects of Rose Bengal (RB)-PDT on the fungus *Trichophyton rubrum* (the most common cause of superficial mycoses) [20]. While this research provided proof of concept for the development of a novel treatment against *T. rubrum* it involved the use of an expensive, high-powered laser making it an unaffordable option to those most at risk. In an effort to produce a low cost alternative we then evaluated the potential of light-emitting diodes (LEDs) within the UVA, UVB and UVC spectra. Our results demonstrated that exposure at 280 nM (UVC) to *T. rubrum* spores at a fluence of 0.5 J/cm^2^ was germicidal [21]. Unfortunately there are obvious carcinogenic risks associated with utilising UVC radiation [22] and as a result we suggested that a more appropriate use of UVC could be in the decontamination of patient shoes, which represent potential reservoirs of re-infection.

The need for a safe, effective and low cost alternative therapy has led to this current study in which the *in vitro* antifungal effects of RB-PDT using an inexpensive green (530 nm) LED, as a versatile light source, were demonstrated. We tested our PDT regime as both a stand-alone therapy and as a component of combinational therapy with a number of clinically important antifungal drugs (clotrimazole and terbinafine) [11]. The findings of this study have the potential to dramatically reduce conventional treatment times, increasing both affordability and patient compliance.

## Results and discussion

We previously demonstrated the antifungal effects of PDT using 140 μM RB activated using a high-powered green laser using a fluence of 228 J/cm^2^ on *T. rubrum* spore suspensions [20]. *T. rubrum* is a clinically significant pathogen causing up to 69.5% of all human dermatophytosis [23]. In an effort to develop an alternative, low cost treatment without the need for expensive lasers our present study investigates the potential of integrating LEDs as part of a standalone RB-PDT regime and as a combinational therapy with currently used antifungal drugs.

### Photodynamic activation of RB using a green LED has a fungicidial effect on *T. rubrum* spores

**Figure 1:**
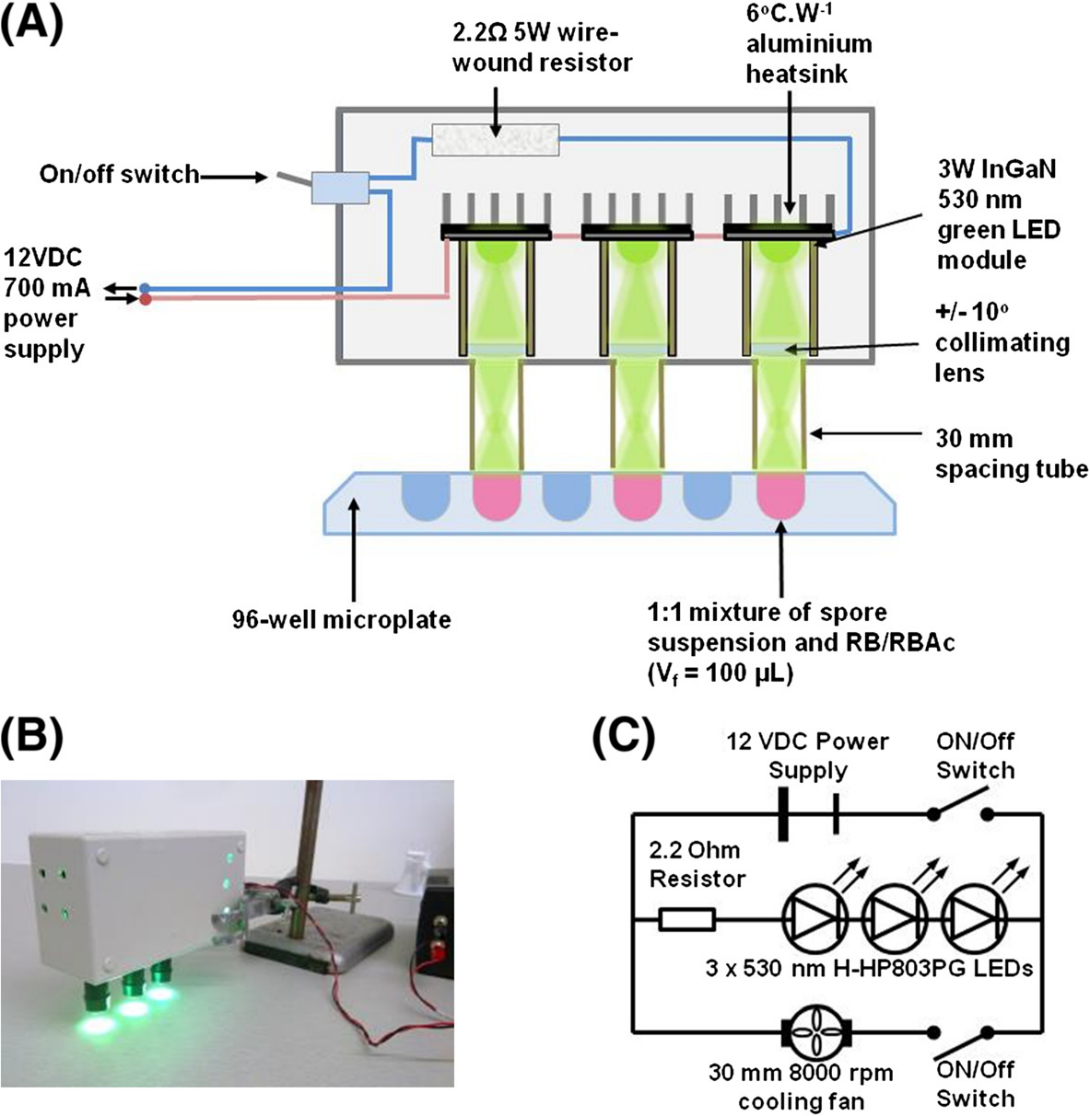
**The 530 nm LED lamp system developed for PDT in this study. (A)** Internal schematic view of the green LED lamp designed and constructed by the author for RB-PDT irradiation of *T. rubrum* spores. **(B)** LED lamp under operation (bottom left). **(C)** Circuit diagram of electrical components comprising the LED lamp (bottom right).

**Figure 2:**
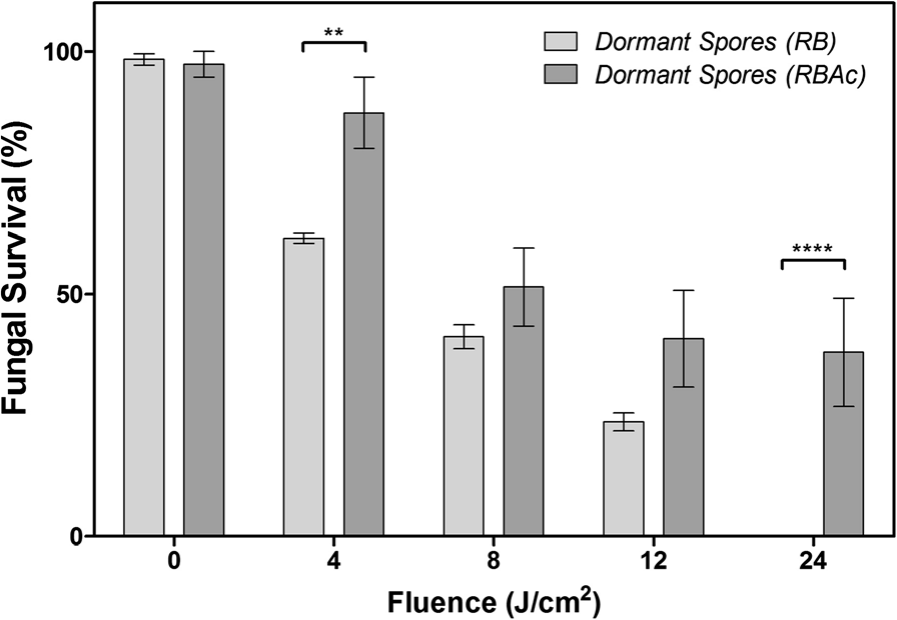
**Fungicidal activity of photodynamic treatment using LED lights at 530 nm and either Rose Bengal or Rose Bengal diacetate on resting spores of** ***T. rubrum*** **.** Data are mean values and standard error from three replicate experiments; analysed by two-way ANOVA (* p <0.05, ** p <0.01, ***p <0.001).

There are many benefits to using LEDs including their versatility in clinical settings, long lifetimes, safety and low cost. In fact, the light source described in this study has been made from readily available items found at a typical electronics store at a cost of approximately US$50 (Figure 1). Using our RB-PDT regime, *T. rubrum* spore suspensions containing RB were irradiated, using a green LED, for various time periods up to 30 minutes. Following irradiation, spore suspensions were spread onto PDA plates and quantified after incubation to assess fungal viability. Exposure of *T. rubrum* spores at a fluence of 24 J/cm^2^ (or an irradiation exposure time of 30 minutes) resulted in the death of 100% of the fungus (Figure 2). Plates were incubated for a further 8 weeks (post-PDT), during which time they were monitored weekly. Following this incubation period no growth was observed on any of the plates containing irradiated spores, indicating that the mechanism of RB-PDT was not simply fungistatic but fungicidal. In all experiments, irradiation of spores using the LED light only (i.e. without RB) or non-irradiated RB (referred to as dark toxicity) did not reduce *T. rubrum* viability compared with the growth control (See Additional file 1: Tables S1 & S2). The performance of our LED lamp system was vastly superior compared to our previous study using a 532 nm laser. The laser required a much larger fluence of 228 J/cm^2^ and resulted in 85% growth inhibition [14].

### Uptake and intracellular localization of RB is not a critical factor to PDT inhibition of *T. rubrum*

**Figure 3:**
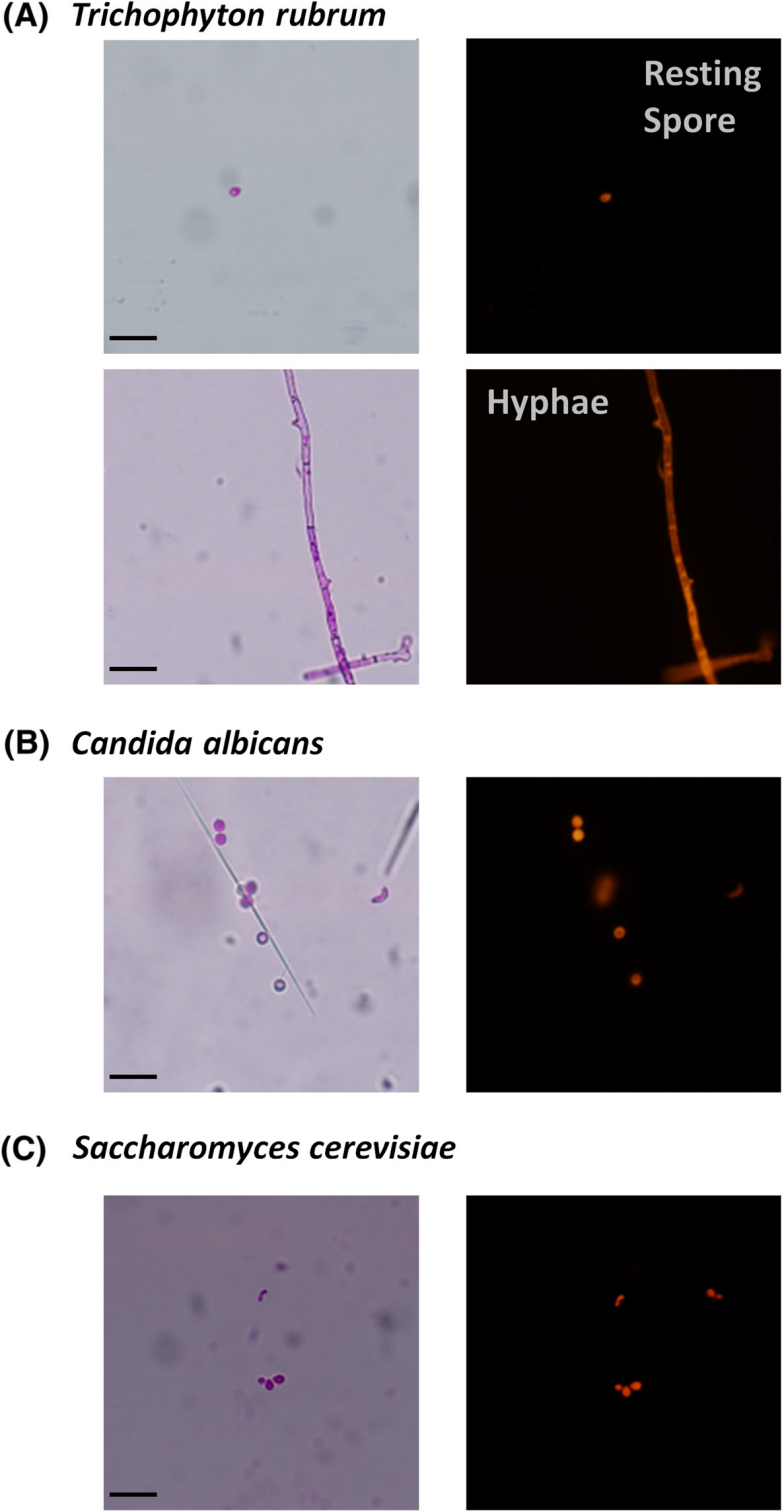
**Micrographs showing the uptake of Rose Bengal by fungi (A) resting spores and hyphae of** ***T. rubrum*** **, (B)** ***C. albicans*** **and (C)** ***S. cerevisiae*** **.** The images were captured under brightfield and fluorescence microscopy at 400 X magnification; the scale bar (Bottom right) is 10 μm.

RB is very soluble in aqueous media and due to its polar nature accumulates very inefficiently within cells, localizing to the outside of fungal spores and hyphae [24,25] (Figure 3). As such its phototoxic effects are restricted to the cell membrane of *T. rubrum* spores and other fungi. In an effort to investigate if it was possible to enhance the potency of RB-PDT we included RB diacetate (RBAc) in our study. RBAc is a modified derivative of RB in which the acetate groups have been added to the xanthene ring making it more hydrophobic and improving its uptake within target cells [14]. A further advantage of the addition of an acetate group is that the photosensitizing property of the compound is quenched, which is highly desirable from a clinical viewpoint as it prevents unwanted photoactivation [24,26]. Following uptake, the acetate groups are removed by endogenous carboxylic esterases, restoring the compound’s functional/photosensitizing capabilities [24].

The spore stage of *T. rubrum* was targeted because the spores are thought to be the primary means by which humans become infected and re-infected [27] and can be used to make standardised inoculum. Spores are the dormant stage of the fungus and have been demonstrated to be more resistant than hyphae to the uptake and inhibitory effects of antifungal agents [28]. However, despite their importance in disease transmission, very little is known regarding the enzymatic activities of dormant *T. rubrum* spores. When we employed RBAc as the photosensitizer in our PDT regime, there was significantly less fungal growth inhibition (68% ±12) compared to that achieved by RB-PDT (99.7% ±0.1) (Figure 2). This result suggested that there were low levels of carboxylic esterase activity associated with *T. rubrum* spores and as a result the remaining studies were carried out using RB.

### RB-PDT displays broad-spectrum antifungal inhibition and is effective against dermatophytic fungi and yeasts

RB has previously been demonstrated to kill a number of pathogenic microoganisms including protozoa [29], Gram-positive bacteria [30] and *T. rubrum* [20]. In the current study the effect of our PDT regime was also tested on the pathogenic yeast *Candida albicans* and the model fungus *Saccharomyces cerevisiae*. The most common cause of fungal disease is *C. albicans*, which causes a range of conditions from superficial infection of mucous membranes to potentially fatal invasive infections and septicaemia [31]. An emerging issue with infections caused by *Candida* spp. is drug resistance with an increasing frequency of azole resistance occurring in clinical isolates [32]. This has driven the need for the development of additional and novel antifungals to combat this pathogenic fungus. A further complication with *Candida* spp. is their ability to form biofilms not only when causing infection but on medical devices [18].

**Figure 4:**
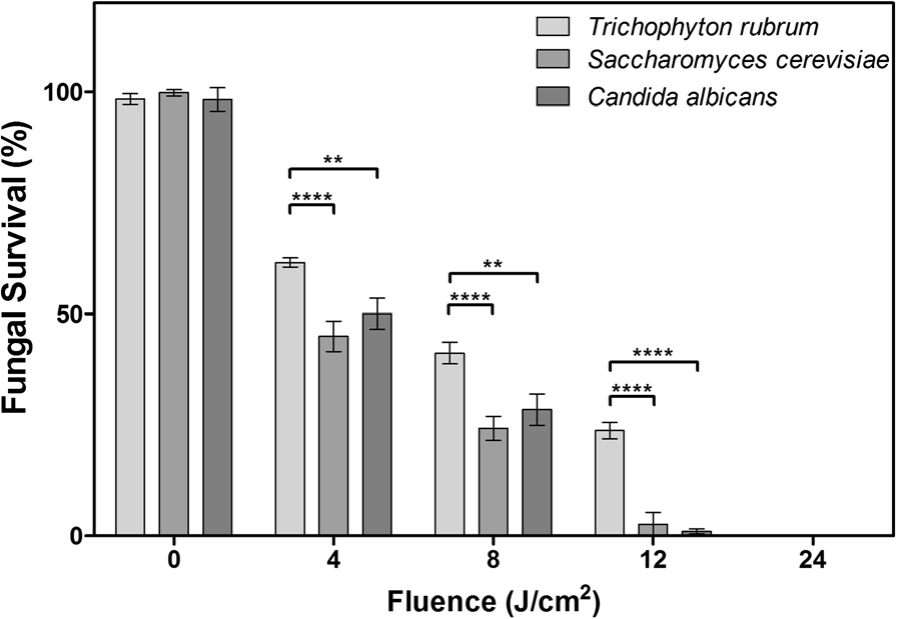
**Fungicidal activity of photodynamic treatment using LED lights at 530 nm and Rose Bengal on the pathogenic fungi** ***T. rubrum, C. albicans*** **and** ***S. cerevisiae*** **.** Data are mean values and standard error from three replicate experiments; analysed by two-way ANOVA (* p <0.05, ** p <0.01, ***p <0.001).

*S. cerevisiae* and *C. albicans* were both sensitive to our RB-PDT regime further highlighting the broad-spectrum capabilities of RB-PDT against fungi (Figure 4). Both *S. cerevisiae* and *C. albicans* were significantly more sensitive to RB-PDT than *T. rubrum* spores (Figure 4). This indicates that RB-PDT could be a potent therapeutic option for a number of pathogenic fungi. Furthermore, a recent study has also demonstrated the broad-spectrum inhibitory effectiveness of RB-PDT at reducing the growth of the pathogenic fungi *Fusarium solani*, *Aspergillus fumigatus* and *C. ablicans* [33]. Studies utilising different PDT protocols and different photosensitisers, e.g. methylene blue and photogem, have also reported promising results against *C. albicans* that indicate that PDT can act much more quickly than antifungal drugs [18,34]. However, some studies have reported varied success with *Candida* spp., noting that biofilms exhibited greater resistance to PDT [18]. The obvious potential of PDT has led to the early development of patch delivery systems for the treatment of oral Candidiasis. Experiments in murine models used adhesive patches to apply the PS photogem to the oral cavity; this was followed by exposure to a red LED light resulting in significant reductions in *C. albicans* viability [35].

### RB-PDT is potentially effective as a combination therapeutic

The use of combination drug strategies is well established in mycology, particularly in treating infections caused by *Candida* spp. [28]. However, there is very little information available on whether antifungal PDT could be ulitised as an adjunct therapy with antifungal drugs. Prior to combining antifungal therapy with RB-PDT we first needed to establish the MIC of our clinical isolate to three commonly used antifungal agents, clotrimazole (CLT), miconazole (MCZ) and terbinafine (TRB). Both CLT and MCZ are over-the-counter (OTC) topical antifungal azoles, while TRB, an allylamine, is the mainstay of orally administered treatments for onychomycosis [4,9]. Using a modified microdilution method, based on the Sensititre YeastOne™ assay (Thermo Fisher, Australia), the MICs for CLT, MCZ and TRB for *T. rubrum* were 0.5 μg/mL, 0.2 μg/mL and 0.01 μg/mL respectively. Our combination therapy protocol involved pre-incubation of spores at sub-inhibitory concentrations of either CLT (0.1 μg/mL), or MCZ (0.1 μg/mL), or TRB (0.005 μg/mL) at 30°C for 72 hours prior to undergoing our RB-PDT regime. These sub-inhibitory concentrations were five and two times lower than the MICs of each of the respective drugs alone.

**Figure 5:**
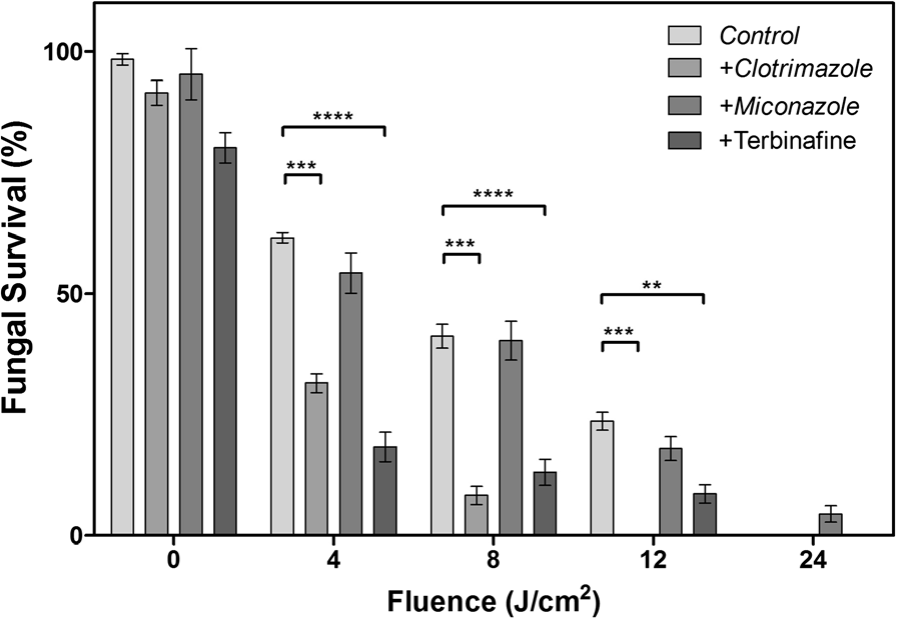
**Fungicidal activity of drug treatment combined with photodynamic treatment using LED lights at 530 nm and Rose Bengal on** ***T. rubrum*** **.** Data are mean values and standard error from three replicate experiments; analysed by two-way ANOVA (* p <0.05, ** p <0.01, ***p <0.001).

The results of our combined antifungal drug/RB-PDT regime are shown in Figure 5. Exposure of spores to pre-treatment with CLT followed by RB-PDT resulted in the most dramatic reduction of lethal dose of PDT from 24 J/cm^2^ to 12 J/cm^2^ compared to non-drug treated controls (i.e. lowering the time to achieve 100% kill from 30 minutes to 15 minutes). A significant though, less pronounced, inhibitory effect was demonstrated when a sub-inhibitory concentration of TRB was used. In contrast, a combination of sub-lethal MCZ (0.1 μg/mL) followed by RB-PDT did not show enhanced inhibition, producing a similar dose–response curve to the non-drug-treated controls, which were also subjected to RB-PDT. This was surprising considering the findings of a number of groups demonstrated that the antifungal MCZ actually induced ROS production in both *Candida* and *S. cerevisae* [36,37]. In fact, Snell *et al*., suggested that MCZ could be used to increase the efficacy of PDT. We did not see a similar increase in efficacy/sensitivity under our PDT regime although it should be noted we used a much lower concentration of MCZ, 0.1 μg/mL as compared to 25 μg/mL [37].

Interestingly, a modest increase in the dark toxicity of RB (i.e. in the absence of light activation) was noted when *T. rubrum* spores were pre-incubated at sub-inhibitory concentrations of the aforementioned drugs and allowed to germinate followed by incubation in 140 μM RB (i.e. not photoactivated) (See Additional file 1: Table S3). This increase in dark toxicity suggests that even at sub-lethal doses, these drugs were capable of weakening/destabilising the cell membrane of germinated spores/hyphae enabling an increased uptake/penetration of RB. It is important to note that neither 140 μM RB nor sub-lethal concentrations of CLT, MCZ and TRB individually, under the same experimental conditions, had an inhibitory effect on the viability of *T. rubrum*. Thus the presence of sub-lethal drug doses of CLT or TRB resulted in enhanced sensitivity to RB-PDT compared to non-drug treated controls. A similar finding was also observed after pretreatment of *C. ablicans* with saponin, a compound capable of creating pores in fungal membranes that significantly increased the uptake and photodynamic effects of RB [38]. Other pretreatment and PDT regimes have utilised specific inhibitors of cellular functions such as antimycin A which was shown to synergistically increase the efficacy of PDT against *Candida* spp. [39].

It has been demonstrated that PDT using methylene blue caused increased membrane permeability in *C. albicans* making it more susceptible to drug treatment [40]. The same authors suggested that this could be a useful strategy in overcoming problems with drug resistance issues in *C. albicans*. However, when we changed the order of our combination protocol, first subjecting *T. rubrum* spores to sub-inhibitory doses of RB-PDT (fluence of 12 J/cm^2^) followed by drug treatment with CLT, MCZ, TRB and using standardised YO10 YeastOne Sensititre plates (Thermo Fisher, Australia), we noted no reduction in the MICs for any of the nine antifungal drugs tested (See Additional file 1: Table S4).

### Future potential and clinical relevance

While these results are based on *in vitro* spore suspensions and thus their clinical relevance has yet to be evaluated, they are very encouraging and merit further investigation. Our results using sub-inhibitory doses of drug are significant when one considers that the recommended treatment dose of CLT (Canesten® Bayer) is 10 mg/mL/daily for up to four weeks to treat superficial and mucosal infections. The recommend oral dose of TRB (Lamisil®, Novartis) is 250 mg/day for 2 weeks when treating tinea pedis and continuous dosing (250 mg/day) for a minimum of 12 weeks when treating for toenail onychomycosis [9,36]. Interestingly, combination therapy using PDT and a suboptimal antibiotic treatment in a mouse wound model, infected with a virulent strain of the bacterium *Pseudomonas aeruginosa*, demonstrated a synergistic therapeutic effect that was capable of curing 60% of mice of this fatal infection [41].

The use of RB in humans has recently received a lot of attention due to its remarkable potential in cancer therapy [42,43]. However in contrast to our antifungal PDT therapy, RB isn’t used in combination with PDT, it is directly injected into the lesion as a 10% solution (PV-10) in a procedure known as intralesional (IL) PV-10 therapy. RB appears to be selectively cytotoxic to tumour cells while sparing any neighbouring healthy cells. Additionally, PV-10 was found to enhance the host’s systemic immunity to fight the tumours [42]. A topical form of PV-10, termed PH-10 (0.002-0.01% Rose Bengal), has successfully completed phase 2 clinical trials for psoriasis and atopic dermatitis with encouraging results on human subjects (clinicaltrials.gov). Therefore RB may be approved for use in humans allowing for greater development of PDT as a treatment for dermatophytoses.

## Conclusions

We believe the RB-PDT approach described in this study holds considerable potential, directed either as a standalone therapy (particularly where the use of antifungals is contra-indicated) or implemented as an adjunct to conventional antifungal therapy to treat dermatophytoses caused by *T. rubrum* and possibly other fungal species. The use of both green light and RB in treating skin infections is preferential, due to their minimal penetration, restricting phototoxicity to the site of infection [44]. Furthermore, the enhanced antifungal effects of RB-PDT when combined with antifungal agents such as clotrimazole and terbinafine as shown in this study may allow for a reduction in treatment times and costs as well as improving patient compliance.

## Methods

### Cultures and media

Clinical isolates of *T. rubrum* 09-043-3609 and *Candida albicans* 01-132-1299 were provided by Westmead Hospital (medical mycology collection, Westmead Hospital, NSW, Australia), these were maintained on potato dextrose agar (PDA). *Saccharomyces cerevisiae* strain BY4743 was obtained from Thermo Fisher. The *T. rubrum* isolate was incubated for fourteen days at 30°C. Following incubation spores were collected by brushing the mould surface with 5 mL 0.05% Tween 80 solution using a sterile glass rod. The resultant spore suspension was filtered using a 40 μM filter (BD, Franklin Lakes, NJ, USA) to remove hyphal fragments. A haemocytometer was used to adjust the suspension density to ~3 × 10^6^ cfu/mL using sterile phosphate buffered saline (PBS) (Oxoid, Thebarton, Australia).

### Construction of light source

The light source was designed and constructed from basic electronic components to create an effective light source emitting narrow-bandwidth green light. To enable the simultaneous irradiation of replicates, light output was provided by using three 3-watt H-HP803PG LED modules (Roithner LaserTechnik, Austria). Each module consisted of a single emitter (producing green light at a dominant wavelength of 530 nm) pre-mounted on a non-conductive 20 mm hexagonal base. LED modules were wired in series to a 2.2 Ω, 5 watt wire-wound resistor. The entire assembly was enclosed in a rigid ABS plastic casing (sealed, except for circular holes for fan ventilation and light projection) to enable practical use and protect the components from physical handling. To provide further cooling and thermal stability for the LEDs, a 12 V, 8000 rpm, 30 mm cooling fan was connected in parallel to the LED series circuit, and for passive cooling, a 6°C/W, aluminium pin-type heatsink was attached to the base of each LED module using double-sided thermal tape (Figure 1). The directional output of each LED was focused into a narrow beam by attaching a 10° lens collimator (internal to the casing) and 30 mm-length polymer tubing (external to the casing) to the emitting side of each module using epoxy putty and cyanoacrylate adhesive (Selleys, Australia). This arrangement changed the light beam pattern to a circular spot size of 0.785 cm^2^ and confined the light output of each LED to a single well of a black 96-well microplate (Greiner Bio-One, Frickenhausen, Germany), eliminating cross-interference with other wells (Figure 1). The irradiance of each LED was measured at 13.4 mW/cm^2^ using a laser power meter (GentecEO, Canada). Heat transfer from the green LED lamp to the sample and surrounding environment was measured, using a K-type thermocouple connected to a digital multimeter (YH-103, YH, China), to ensure that any resulting inhibitory effect evident following RB-PDT was not the result of photothermal effects produced as a result of the lamp system itself (See Additional file 1: Table S5).

### Photodynamic treatment

A stock solution of Rose Bengal (RB) (Sigma Aldrich, Castle Hill, Australia) was prepared at a concentration of 280 μM in PBS (pH 7.4). The solution was filter sterilized using a 0.22 μm filter (Millipore, Billerica, MA, USA) and wrapped in aluminium foil to prevent unwanted photoactivation. Sample preparation consisted of the addition of 50 μL of 280 μM rose bengal and 50 μL of spore suspension (3 × 10^6^ cfu/mL), specifically microconidia, to wells of a black 96-well microplate. The plate was covered in aluminium foil and incubated with shaking at 150 rpm for 30 minutes at 30°C. Samples were irradiated separately using the LED lamp system for 0, 5, 10, 15 and 30 minutes respectively under aseptic conditions in a laminar flow hood. In the untreated control (RB-L-) and photothermal (RB-L+) treatments, RB was replaced by PBS in both circumstances and irradiated as above.

Fungal viability following RB-PDT was determined by diluting the samples 1:10 with PBS and 50 μL aliquots of the diluted sample was spread onto PDA plates (three plates per sample) then incubated at 30°C for 96 hours. After incubation, colonies were enumerated and the viability was expressed as the ratio of the treated cells relative to untreated cells. Independent experiments were performed at least three times and the results subjected to statistical analysis.

### Susceptibility of *T. rubrum* to clotrimazole, miconazole and terbinafine

The MICs of the antifungal drugs clotrimazole, miconazole and terbinafine (Sigma-Adrich, Castle-Hill, Australia) were determined using a microdilution method incorporating alamarBlue® (Life Technologies, Mulgrave, Australia) as a viability indicator. This method was similar to the standardised YO10 YeastOne Sensititre Plate System (Thermo Fisher, Australia). Stock solutions of miconazole and terbinafine-HCl were prepared in a glass vial by dissolving the drug in DMSO (dimethyl sulfoxide) to attain a final drug concentration of 10 mg/mL. Clotrimazole was prepared to a stock concentration of 5 mg/mL using an identical method. All stock solutions were stored at −20°C.

*S*amples were prepared by sequentially adding 200 μL of 10x alamarBlue® stock solution, 20 μL of *T. rubrum* spore suspension (3 × 10^6^ cfu/mL), 780 μL YeastOne broth (Thermo Fisher, Australia) and mixed evenly. 50 μL of this mixture was pipetted into each well of a 96-well plate containing the required drug ranges. Drugs were prepared by serial dilution to achieve drug concentrations of 0.01 μg/mL and 0.5 μg/mL for clotrimazole and miconazole, and 0.001 to 0.005 μg/mL for terbinafine. The 96-well plate was sealed with a lid and polyethylene film to prevent evaporation; the plate was incubated at 30°C for 96 hours with shaking. Colour changes (purple or pink) were read visually. The MICs of both drugs were determined to be the lowest drug concentration in which no growth occurred. Independent experiments were performed at least three times and the results subjected to statistical analysis.

### Combination therapy

Our combination therapy consisted of exposure to a sublethal dose of antifungal agent followed by RB-PDT. Assays were performed by combining 500 μL of a *T. rubrum* spore suspension (3 × 10^6^ cfu/mL) with 400 μL YeastOne broth (Thermo Fisher, Australia) followed by the addition of either clotrimazole (final concentration 0.1 μg/mL), miconazole (final concentration 0.1 μg/ml) or terbinafine-HCl (final concentration 0.005 μg/mL) to a final volume of 1.0 mL. Samples were incubated for 72 hours at 30°C. Independent experiments were performed at least three times and the results subjected to statistical analysis. Following the incubation period, drug-treated spore suspensions, consisting of germinated spores/hyphae following the 72 hours growth period, were subjected to RB-PDT (described earlier). Post-PDT samples were diluted 1:5 and 50 μL aliquots per sample were plated onto three PDA plates. The plates were incubated at 30°C for 96 hours and the percentage survival determined for each drug-PDT treatment by enumerating the cfu/mL.

### Statistical analysis

Quantitative data for the effects of PDT were compared by two-way ANOVA between relevant treatments using Graphpad Prism Version 5.02 for Windows (Graphpad Software, San Diego, CA, USA).

## Additional file

Additional file 1: Table S1. Dark Toxicity of 140 μM of Rose Bengal tested against different fungi. **Table S2.** Effect of exposure to light from the LED system on fungal viability. **Table S3.** Dark toxicity of 140 μM Rose Bengal following a 72 hr/30°C incubation of *T. rubrum* spores in sub-inhibitory concentrations of a number of antifungal drugs: clotrimazole (CTL: 0.1 μg/ml); miconazole (MCZ: 0.1 μg/ml); and terbinafine hydrochloride (TRB: 0.005 μg/ml) (n = 3). **Table S4.** Changes in drug MIC against *Trichophyton rubrum* following RB-PDT (140 μM Rose Bengal and 12 J/cm^2^). **Table S5.** Measurement of heating effects due to the LED system during activation.

## Abbreviations

PDA: Potato dextrose agar
RB: Rose Bengal
PDT: Photodynamic therapy
LED: Light-emitting diode
CFU: Colony forming unit
CLT: Clotrimazole
TRB: Terbinafine
MCZ: Miconazole

## References

[1] Burmester A, Shelest E, Glockner G, Heddergott C, Schindler S, Staib P, Heidel A, Felder M, Petzold A, Szafranski K, Feuermann M, Pedruzzi I, Priebe S, Groth M, Winkler R, Li W, Kniemeyer O, Schroeckh V, Hertweck C, Hube B, White TC, Platzer M, Guthke R, Heitman J, Wöstemeyer J, Zipfel PF, Monod M, Brakhage AA (2011). Comparative and functional genomics provide insights into the pathogenicity of dermatophytic fungi. Genome Biol.

[2] Peres NT, Maranhao FC, Rossi A, Martinez-Rossi NM (2010). Dermatophytes: host-pathogen interaction and antifungal resistance. An Bras Dermatol.

[3] Nenoff P, Kruger C, Ginter-Hanselmayer G, Tietz HJ (2014). Mycology - an update. Part 1: Dermatomycoses: causative agents, epidemiology and pathogenesis. J Deutschen Dermatol Gesellschaft = J German Soc Dermatol: JDDG.

[4] Crawford F, Hollis S (2007). Topical treatments for fungal infections of the skin and nails of the foot. Cochrane Database Syst Rev.

[5] Meis JF, Verweij PE (2001). Current management of fungal infections. Drugs.

[6] El-Gohary M, van Zuuren EJ, Fedorowicz Z, Burgess H, Doney L, Stuart B, Moore M, Little P (2014). Topical antifungal treatments for tinea cruris and tinea corporis. Cochrane Database Syst Rev.

[7] Crowley PD, Gallagher HC (2014). Clotrimazole as a pharmaceutical: past, present and future. J Appl Microbiol.

[8] Prabagar B, Yoo BK, Woo JS, Kim JA, Rhee JD, Piao MG, Choi HG, Yong CS (2007). Enhanced bioavailability of poorly water-soluble clotrimazole by inclusion with beta-cyclodextrin. Arch Pharm Res.

[9] Bell-Syer SE, Khan SM, Torgerson DJ (2012). Oral treatments for fungal infections of the skin of the foot. Cochrane Database Syst Rev.

[10] Odds FC, Brown AJ, Gow NA (2003). Antifungal agents: mechanisms of action. Trends Microbiol.

[11] Piscitelli SC, Rodvold KA, Pai MP (2011). Drug Interactions in Infectious Diseases.

[12] Ting PC, Walker SS (2008). New agents to treat life-threatening fungal infections. Curr Top Med Chem.

[13] Niewerth M, Korting HC (1999). Management of onychomycoses. Drugs.

[14] Panzarini E, Inguscio V, Dini L (2011). Timing the multiple cell death pathways initiated by Rose Bengal acetate photodynamic therapy. Cell Death Dis.

[15] Dougherty TJ (2002). An update on photodynamic therapy applications. J Clin Laser Med Surg.

[16] Calzavara-Pinton PG, Venturini M, Sala R (2005). A comprehensive overview of photodynamic therapy in the treatment of superficial fungal infections of the skin. J Photochem Photobiol B.

[17] Lyon JP, Moreira LM, de Moraes PC, dos Santos FV, de Resende MA (2011). Photodynamic therapy for pathogenic fungi. Mycoses.

[18] Calzavara-Pinton P, Rossi MT, Sala R, Venturini M (2012). Photodynamic antifungal chemotherapy. Photochem Photobiol.

[19] Dai T, Fuchs BB, Coleman JJ, Prates RA, Astrakas C, St Denis TG, Ribeiro MS, Mylonakis E, Hamblin MR, Tegos GP (2012). Concepts and principles of photodynamic therapy as an alternative antifungal discovery platform. Front Microbiol.

[20] Cronin L, Moffitt M, Mawad D, Morton OC, Lauto A, Stack C (2014). An in vitro study of the photodynamic effect of rose bengal on trichophyton rubrum. J Biophotonics.

[21] Cronin LJ, Mildren RP, Moffitt M, Lauto A, Morton CO, Stack CM (2014). An investigation into the inhibitory effect of ultraviolet radiation on Trichophyton rubrum. Lasers Med Sci.

[22] De Flora S (2013). Genotoxicity and carcinogenicity of the light emitted by artificial illumination systems. Arch Toxicol.

[23] Chen BK, Friedlander SF (2001). Tinea capitis update: a continuing conflict with an old adversary. Curr Opin Pediatr.

[24] Bottiroli G, Croce AC, Balzarini P, Locatelli D, Baglioni P, Lo Nostro P, Monici M, Pratesi R (1997). Enzyme-assisted cell photosensitization: a proposal for an efficient approach to tumor therapy and diagnosis. The rose bengal fluorogenic substrate. Photochem Photobiol.

[25] Croce AC, Supino R, Lanza KS, Locatelli D, Baglioni P, Bottiroli G (2002). Photosensitizer accumulation in spontaneous multidrug resistant cells: a comparative study with Rhodamine 123, Rose Bengal acetate and Photofrine. Photochem Photobiol Sci: Offic J Eur Photochem Assoc Eur Soc Photobiol.

[26] Bottone MG, Soldani C, Fraschini A, Alpini C, Croce AC, Bottiroli G, Pellicciari C (2007). Enzyme-assisted photosensitization with rose Bengal acetate induces structural and functional alteration of mitochondria in HeLa cells. Histochem Cell Biol.

[27] Coelho LM, Aquino-Ferreira R, Maffei CM, Martinez-Rossi NM (2008). In vitro antifungal drug susceptibilities of dermatophytes microconidia and arthroconidia. J Antimicrob Chemother.

[28] Evans EG (2001). The rationale for combination therapy. Br J Dermatol.

[29] Cruz FS, Lopes LA, De Souza W, Moreno SN, Mason RP, Docampo R (1984). The photodynamic action of rose bengal on Trypanosoma cruzi. Acta Trop.

[30] Dahl TA, Midden WR, Neckers DC (1988). Comparison of photodynamic action by Rose Bengal in gram-positive and gram-negative bacteria. Photochem Photobiol.

[31] Lyon JP, de Resende MA (2006). Correlation between adhesion, enzyme production, and susceptibility to fluconazole in Candida albicans obtained from denture wearers. Oral Surg Oral Med Oral Pathol Oral Radiol Endod.

[32] Cuenca-Estrella M (2014). Antifungal drug resistance mechanisms in pathogenic fungi: from bench to bedside. Clin Microbiol Infect.

[33] Arboleda A, Miller D, Cabot F, Taneja M, Aguilar MC, Alawa K, Amescua G, Yoo SH, Parel JM (2014). Assessment of rose bengal versus riboflavin photodynamic therapy for inhibition of fungal keratitis isolates. Am J Ophthalmol.

[34] Dovigo LN, Pavarina AC, Mima EG, Giampaolo ET, Vergani CE, Bagnato VS (2011). Fungicidal effect of photodynamic therapy against fluconazole-resistant Candida albicans and Candida glabrata. Mycoses.

[35] Mima EG, Pavarina AC, Dovigo LN, Vergani CE, Costa CA, Kurachi C, Bagnato VS (2010). Susceptibility of Candida albicans to photodynamic therapy in a murine model of oral candidosis. Oral Surg Oral Med Oral Pathol Oral Radiol Endod.

[36] Teichert MC, Jones JW, Usacheva MN, Biel MA (2002). Treatment of oral candidiasis with methylene blue-mediated photodynamic therapy in an immunodeficient murine model. Oral Surg Oral Med Oral Pathol Oral Radiol Endod.

[37] Snell SB, Foster TH, Haidaris CG (2012). Miconazole induces fungistasis and increases killing of Candida albicans subjected to photodynamic therapy. Photochem Photobiol.

[38] Coleman JJ, Okoli I, Tegos GP, Holson EB, Wagner FF, Hamblin MR, Mylonakis E (2010). Characterization of plant-derived saponin natural products against Candida albicans. ACS Chem Biol.

[39] Chabrier-Rosello Y, Giesselman BR, De Jesus-Andino FJ, Foster TH, Mitra S, Haidaris CG (2010). Inhibition of electron transport chain assembly and function promotes photodynamic killing of Candida. J Photochem Photobiol B.

[40] Giroldo LM, Felipe MP, de Oliveira MA, Munin E, Alves LP, Costa MS (2009). Photodynamic antimicrobial chemotherapy (PACT) with methylene blue increases membrane permeability in Candida albicans. Lasers Med Sci.

[41] Lu Z, Dai T, Huang L, Kurup DB, Tegos GP, Jahnke A, Wharton T, Hamblin MR (2010). Photodynamic therapy with a cationic functionalized fullerene rescues mice from fatal wound infections. Nanomedicine (Lond).

[42] Toomey P, Kodumudi K, Weber A, Kuhn L, Moore E, Sarnaik AA, Pilon-Thomas S (2013). Intralesional injection of rose bengal induces a systemic tumor-specific immune response in murine models of melanoma and breast cancer. PLoS One.

[43] Tan CY, Neuhaus SJ (2013). Novel use of Rose Bengal (PV-10) in two cases of refractory scalp sarcoma. ANZ J Surg.

[44] Wachter E, Dees C, Harkins J, Scott T, Petersen M, Rush RE, Cada A (2003). Topical rose bengal: pre-clinical evaluation of pharmacokinetics and safety. Lasers Surg Med.

