# Supplementary material for: In vitro combination therapy using low dose clotrimazole and photodynamic therapy leads to enhanced killing of the dermatophyte Trichophyton rubrum
Source: BMC Microbiol. 2014 Oct 15;14:261. doi: 10.1186/s12866-014-0261-z (PMC4210577; doi:10.1186/s12866-014-0261-z)
Supplement: Additional file 1: Table S1. — Dark Toxicity of 140 μM of Rose Bengal tested against different fungi. Table S2. Effect of exposure to light from the LED system on fungal viability. Table S3. Dark toxicity of 140 μM Rose Bengal following a 72 hr/30°C incubation of T. rubrum spores in sub-inhibitory concentrations of a number of antifungal drugs: clotrimazole (CTL: 0.1 μg/ml); miconazole (MCZ: 0.1 μg/ml); and terbinafine hydrochloride (TRB: 0.005 μg/ml) (n = 3). Table S4. Changes in drug MIC against Trichophyton rubrum following RB-PDT (140 μM Rose Bengal and 12 J/cm2). Table S5. Measurement of heating effects due to the LED system during activation. [file 12866_2014_261_MOESM1_ESM.docx]

Table S1 Dark Toxicity of 140 µM of Rose Bengal tested against different fungi.

| Organism | Fungal viability (%)* |
| --- | --- |
| *Trichophyton rubrum* | 98.3 % ± 2.1 |
| *Candida albicans* | 98.2% ± 4.5 |
| *Saccharomyces cerevisiae* | 99.7% ± 1.2 |

*Determined as a percentage of an untreated control

Table S2 Effect of exposure to light from the LED system on fungal viability.

| Irradiation Time (min) | Fungal Viability (%)* |
| --- | --- |
| 0 | 100 |
| 5 | 100.6 % ± 5.1 |
| 10 | 98.7 % ± 5.9 |
| 15 | 102.3 % ± 6.0 |
| 30 | 99.8 % ± 5.8 |

*Determined as a percentage of an untreated control

Table S3 Dark toxicity of 140 μM Rose Bengal following a 72hr/30°C incubation of *T. rubrum* spores in sub-inhibitory concentrations of a number of antifungal drugs: clotrimazole (CTL: 0.1 μg/ml); miconazole (MCZ: 0.1 μg/ml); and terbinafine hydrochloride (TRB: 0.005 μg/ml) (n = 3).

| Treatment | RB-L-CLT+ | RB+L-CLT+ | RB-L-MCZ+ | RB+L-MCZ+ | RB-L-TRB+ | RB+L-TRB+ |
| --- | --- | --- | --- | --- | --- | --- |
| RB conc (μM) | 0 | 140 | 0 | 140 | 0 | 140 |
| Viability (%)* | 100 | 91.5 | 100 | 95.5 | 100 | 80.1 |
| SD (%) | 0 | 4.4 | 0 | 7.4 | 0 | 5.4 |

*Determined as a percentage of an antifungal drug treated control

Table S4 Changes in drug MIC against *Trichophyton rubrum* following RB-PDT (140 µM Rose Bengal and 12 J/cm^2^).

| Drug | MIC (µg/ml) | ΔMIC Following PDT |
| --- | --- | --- |
| Micafungin* | > 8 | No change |
| Caspofungin* | 0.06 | No change |
| 5-flucytosine* | > 64 | No change |
| Posaconazole* | 0.015 | No change |
| Voriconazole* | < 0.008 | No change |
| Itraconazole* | < 0.015 | No change |
| Fluconazole* | 1 | No change |
| Anidulafungin* | 0.12 | No change |
| Amphotericin B* | 0.5 | No change |
| Miconazole | 0.2 | No change |
| Clotrimazole | 0.5 | No change |
| Terbinafine | 0.01 | No change |

*Tested using Sensititre YO10 plate

Table S5 Measurement of heating effects due to the LED system during activation.

| Time (min) | 0 | 15 | 30 | 45 | 60 |
| --- | --- | --- | --- | --- | --- |
| Intake Air Temperature (°C) | 21 | 22 | 22 | 22 | 22 |
| Exhaust Air Temperature (°C) | 21 | 27 | 35 | 35 | 36 |
| Sample Temperature (°C) | 22 | 23 | 23 | 23 | 23 |
